# Rosemary and neem methanolic extract: antioxidant, cytotoxic, and larvicidal activities supported by chemical composition and molecular docking simulations

**DOI:** 10.3389/fpls.2023.1155698

**Published:** 2023-05-18

**Authors:** Haifa A. S. Alhaithloul, Mesfer M. Alqahtani, Mohamed A. Abdein, Mohamed A. I. Ahmed, Abd El-Latif Hesham, Mohammad M. E. Aljameeli, Reem N. Al Mozini, Fatehia N. Gharsan, Suzan M. Hussien, Yasser A. El-Amier

**Affiliations:** ^1^ Biology Department, College of Science, Jouf University, Sakaka, Saudi Arabia; ^2^ Department of Biological Sciences, Faculty of Science and Humanities, Shaqra University, Ad-Dawadimi, Saudi Arabia; ^3^ Department of Biology, Faculty of Science and Arts, Northern Border University, Rafha, Saudi Arabia; ^4^ Plant Protection Department, Faculty of Agriculture, Assiut University, Assiut, Egypt; ^5^ Genetics Department, Faculty of Agriculture, Beni-Suef University, Beni-Suef, Egypt; ^6^ Department of Biology, College of Science and Arts, Qassim University, Unaizah, Saudi Arabia; ^7^ Biology Department, Faculty of Science, Al-Baha University, Al-Baha, Saudi Arabia; ^8^ Botany Department, Faculty of Science, Mansoura University, Mansoura, Egypt

**Keywords:** rosemary, neem, GC/MS, antioxidant, larvicidal activity, docking, antimicrobial, *T. castaneum*

## Abstract

This study aimed to employ GC–MS to assess the chemical composition of MeOH leaf extracts of *R. officinalis* and *A. indica* and evaluate their insecticidal, antioxidant, and antibacterial activities. Twelve components, representing 98.61% and 100% of the total volatile compounds, were deduced from the extracted *R. officinalis* and *A. indica*, respectively, using this method. In *R. officinalis* extract, limonene is typically positioned as the main component (23.03%), while the main chemicals identified in *A. indica* extract were methyl (E)-octadec-13-enoate (23.20%) and (2R)-1,3,8-trimethyl-4-propyl-5-ethyl-2-(1-hydroxyethyl)-7-methoxycardonylethyl-6-methylenecarbonyl-porphyrin (23.03%). Both extracts of *R. officinalis* and *A. indica* exhibited different toxicity against the stored grain pest *T. castaneum*, with LC_50_ values of 1.470 and 2.588 mg/ml, respectively. Additionally, after 4 and 5 h of treatment at a concentration of 0.2 mg/ml, the *A. indica* extract showed the highest levels of repellent action (81.4% and 93.4%), and the *R. officinalis* extract showed a good repellent rate (64.9% and 80.7%) against *T. castenum* larvae. With an IC_50_ value of 35.83 and 28.68 mg/L and a radical scavenging activity percentage of 67.76% and 72.35%, the leaf extract was found to be the most potent plant extract when tested for DPPH antioxidant activity. Overall results showed that MeOH extracts of *R. officinalis* and *A. indica* were more effective against *S. aureus* than *E. coli*. To determine how the investigated chemicals attach to the active sites of *E. coli* DNA gyrase A and *S*. *aureus* undecaprenyl diphosphate synthase, docking studies were carried out. The consensus score analysis showed that limonene exhibits the best binding energy with both enzymes in docking analysis and more stability in molecular dynamics simulations. The RMSD was obtained at 20.6 and 4.199 (Kcal/mole). The two compounds were successfully used in molecular dynamics simulation research to generate stable complexes with DNA gyrase A.

## Introduction

The storing of cereals is the most significant post-harvest activity. Insect attacks caused the most losses during storage. The traditional synthetic insecticides used today to protect goods in storage and prevent post-harvest losses typically manage insect pests on stored cereals (Deichmann et al., 2021). However, according to FAO data, 10%–25% of the world’s gathered food is destroyed each year by insects and rodent pests (Gomes et al., 2021).

Reducing dependence on synthetic pesticides and replacing harmful fumigants with natural alternatives that are widely accepted by environmental pollution, health, economics, and value are important (Scully et al., 2021). However, these pesticides come with a number of issues, including toxicity residues in food, worker safety, insect resistance, and treatment costs. It is important to note that employing synthetic insecticides can lead to widespread contamination of the air, water, and food supply, which can have a negative impact on health (Yang et al., 2020). Interestingly, botanical extracts from natural plants primarily contain secondary metabolites and aromatics with a low molecular weight that have chemical defenses against various insects (Morrison et al., 2019).

Investigating floral biodiversity and employing safe insecticides of botanical origin as an easy and sustainable means of mosquito control is one of the most effective alternative methods under the biological control program (Larson et al., 2020). The plant known as rosemary, or *Rosmarinus officinalis*, is a member of the Lamiaceae family and native to the Mediterranean region. The extracts of the leaves can be used to identify a number of phytocompounds with pharmacological properties, and the concentration of these molecules varies in each plant specimen (Tivana et al., 2021).

As a result, reading through the various works on medicinal plants has shown that there are numerous plants with distinct pharmacological characteristics. One of them is *Azadirachta indica*, also known as neem, which is a famous fast-growing evergreen plant from the Meliaceae family (Girish and Shankara, 2008). Numerous studies have demonstrated the antibacterial, antiviral, antifungal, and insecticidal effects of *A. indica* leaf extracts. In view of these plants’ insecticidal efficiencies, they are to be tested against *Tribolium castaneum*.

Hemoproteins called P450s function as terminal oxidases the monooxygenase system. P450, which serves as the substrate binding protein in the P450 monooxygenase system, NADPH-cytochrome P450 reductase (CPR), which transfers electrons from NADPH to CYPs, and cytochrome b5, which transfers electrons from NADH to CYPs as an additional potential electron donor (Hammer et al., 2019). The first insect whose genome has been sequenced is *T. castaneum*, the red flour beetle. These characteristics make *T. castaneum* an ideal insect model for functional genomics, tracing the origins of pesticide resistance, and creating new insecticide targets for pest control (Hameed et al., 2015). The potential chemical interactions between the most concentrated substances and DNA gyrase and UPP were next explored by a molecular docking study.

In this study, we focused on evaluating the repellent and insecticidal repellent effects of hydromethanolic extracts obtained from *R. officinalis* and *A. indica* leaves on stored grain pests. *T. castaneum* larvae to investigate novel plant-derived agents to control stored grain agents. Additionally, we demonstrated the induction of toxicity gene expression in *T*. *castaneum* as a response to the exposure of selected plant extracts and their interactions.

## Materials and methods

### Collecting and rearing of insect

From various contaminated flour, wheat, and barley stored grains found close to Beheira Governorate in Egypt, the insect *T. castaneum* was gathered. The samples were grown at 28°C in sterilized glass jars on 5% wheat flour media with 5% brewer’s yeast. Specific life stages of the *T. castaneum* insect were removed during subculture from the laboratory colony.

### Plant sample collection and preparation of extracts

In March 2021, the Lamiaceae family shrub *R. officinalis* L. and the Meliaceae family tree *A. indica* L. were removed in good health from their respective plantations in the Botanical Gardens and Ornamental Plants Departments (the flowering season). The plant was recognized by Dr. Yasser A. El-Amier of the Department of Plant Ecology at the Faculty of Science at Mansoura University, and voucher specimens (Mans. 121815004 and Mans. 130109005) were deposited in the herbarium of the department. To remove any dust or other residues, the samples were physically cleaned and rinsed three times in distilled water, then dried for three weeks in a dark, air-conditioned room at 25°C, and then crushed into powder. Next, in a Soxhlet apparatus and an ultrasonic application, the dried powder was extracted with hydro-methanol (30:70) for 1 h at 60°C. After the extract was concentrated using a rotary evaporator under reduced pressure, the crude extracts were kept at 4°C until further analysis (Sultana et al., 2009).

### Gas chromatography-mass spectrometry analysis

The chemical composition of the hydromethanolic extracts of the studied plants (*R. officinalis* and *A. indica*) was examined using GC/MS (Agilent Technologies) fitted with a GC (7890B) and MS detector (5977A). The column was a fused silica DB-5 capillary column (30 m long, 0.25 mm internal diameter) coated with polydimethyl-siloxane (0.5 µm film thickness). The oven temperature was programmed to be 50°C for 3 min, then heated by 7°C/min to 250°C and maintained isothermally for 10 min at 250°C. Helium served as the carrier gas, flowing at a rate of 1 ml/min under a constant flow model with an injection volume of 2 ul. Ionization energy was set at 70 eV (de Dobbeleer et al., 2012). It was feasible to determine the chemical make-up of each unique plant material by comparing the mass spectrometry data of the numerous extracted plant components to those of the mass spectrometry databases WILEY 09 and NIST 14. Each detected peak has five potential components, according to the GC–MS study. The probability factors and the primary structure patterns of fragmentation were used to choose one of the several proposed components.

### Insecticidal bioassay test

#### Mortality activity

The effects of 70% methanolic extracts from the mentioned samples on *T. castaneum* larval mortality were compared to a positive control treatment and a 10 g/ml chemical standard, such as malathion. The insecticidal effect was evaluated using filter paper bioassay discs described by Abdel-Sattar et al. (2010). A completely randomized design was used to set up five replications at concentrations of 0.1, 0.2, 0.3, and 0.4 mg/ml for each individual plant hydromethanolic extract (30:70). For each preparation, 0.5 ml was pumped and flowed regularly on a disk of filter paper (Wathmann No. 1) placed in a Petri dish. All treated and control filter paper halves were given 20 min to air dry at room temperature to allow the solvent to evaporate (Engel and Moran, 2013). Twenty-third-instar *T. castaneum* larvae were released in the center of the filter paper, which was parafilm-sealed. The experiment was carried out in the dark at 28°C and 655% R.H. Count the dead larvae at 6, 12, 24, 48, and 72 h following the application. A little probe was used to gently contact the insects, and those who did not respond seemed dead (Seada et al., 2016). The Abbott’s formula was used to calculate the corrected mortality (Abbott, 1925).


$$Larval\;mortality\;\left(\%\right)=\frac{Number\;of\;dead\;larvae}{Number\;of\;treated\;larvae}\;\times \;100$$

#### Repellence activity (filter paper disc bioassay)

The repellent effects of *R. officinalis*, *A. indica*, and the control treatment (70% methanol) on *T. castaneum* larvae were evaluated in contrast to the positive control treatment and a 5 g/ml chemical standard like malathion. Five replications for each respective plant 70% methanolic extract (0.05, 0.1, 0.15, and 0.2 mg/L) were used to evaluate the repellency by filter paper disc bioassay methods (Isman, 2006). The filter paper disc was then treated with the appropriate quantities of hydromethanolic plant extractions, while the control half was treated with an equivalent volume of 70% methanol instead of malathion solution. All treated and control filter paper halves were left to air dry at room temperature for 20 min to evaporate the solvent. Released twenty-third-instar *T. castaneum* larvae on both sides’ centers after fixing the paper disc to the bottom of a Petri dish. The experiment was kept running in a dark room at 28°C and 65% R.H. Repellency percentage was measured based on the number of insects that landed on the treated and negative control halves, respectively, at 1, 2, 3, 4, and 5 h post-exposure. The percent repellency (PR) was calculated by Equation (1).


$$\%\;Repellency\;percentage\;=\frac{N_{c}\;–\;N_{t}}{N_{c}+\;N_{t}}\;\times \;100$$

Nc = the number of insects on the untreated half, and Nt = the number of insects on the treated half, after exposure.

### Molecular response of insect to botanical pesticides

The mode of action of botanical pesticides using the selected plant extracts was determined by studying the transcriptional expression levels for detoxifying genes (CYP 4Q7, CYP 450, and CYP sim) in *T. castenum* larvae exposed to hydromethanolic extracts obtained from studied plants using quantitative real-time polymerase chain reaction (qRT-PCR) analysis (Hammer et al., 2019).

### Total RNA isolation cDNA synthesis from *T. castaneum* larvae

Following the manufacturer’s instructions, total RNA was extracted from 0.1** g** of *T*. *castaneum* third instar larva that had been exposed to 0.2 mg/ml hydromethanolic extracts of *R. officinalis* and *A. indica* and standard chemical insecticide malathion (5 mg/ml) for 1 and 2 h, as well as a control treatment (70% methanol) (Huang et al., 2018).

### qRT-PCR analysis

Using Rotor-Gene 6000 real-time PCR detection equipment, third-instar *T. castenum* larvae treated with various hydromethanolic extracts were subjected to real-time PCR for three insecticidal detoxification genes (Qiagen, Germany). **Table 1** shows the primer sequences for qRT-PCR were developed. The actin gene of *T. castaneum* was employed as an internal control gene since it was consistently expressed in this plant. Using the manufacturer’s instructions, 12.5 µl of 1 µl SYBR Green PCR mix, 1 l sets of each primer, 1** l** (1:10) diluted cDNA template plates, and 9.5 µl of DEPC-treated water were all included in the reaction volume of 25 µl. RT-qPCR was amplified in three steps by incubating for 10 min at 95°C, 40 cycles for 15 s each at 95°C, and then 60 s at 72°C. SigmaPlot version 9.0 software was used to calculate the quantity of expressed genes (Systat Software Inc., USA) (Togawa et al., 2008).

**Table 1:** Primers used in quantitative real-time PCR analysis in *T. castaneum* larval stage treated with 70% extracts of studied plants.

Technique	Target Gene	Sequences (5’–3’)	Annealing °C
qRT-PCR	β Actin F	5′ACACACCAAAATGTGCGACG′3	60 °C
	β Actin R	5′CGGTGGTGGTGAACGAGTAA′3	
	CYP sim-F	CATCCGCAAACACAACAAAC	
	CYP sim-R	CGACTGGTCGCTACACTTCA	
	CYP 450-F	5′ TCAACCGACTGCACCTGTAT′3	
	CYP 450-R	5′GCGTATCTATCAGGGCGACT′3	
	CYP 4Q7-F	AGGACTGCGAGCTGGTTTTA	
	CYP 4Q7-R	CCATTGCTGTCTCTGCGATA	

### Antioxidant DPPH assay

Miguel (2010) reports that the hydroalcoholic extracts from the two plants under research were evaluated for antioxidant activity by scavenging 2,2-diphenyl-1-picrylhydrazyl (DPPH) (Sigma-Aldrich, Germany). To make various amounts of methanolic extract, methanol (70%) was used (5, 10, 20, 30, 40, and 50 mg ml^−1^). The sample solution was added to the DPPH solution at various concentrations (1 mL, 0.135 Mm). The ascorbic acid levels in the examined samples served as a benchmark. Using a UV/Vis spectrophotometer, the absorbance of the samples was measured at a wavelength of 517 nm after 30 min at room temperature and in the dark (SPEKOL 11 spectrophotometer, Analytic Jena AG, Jena, Germany). The percentages of antioxidant scavenging activities were calculated using the following equation, using a DPPH solution in methanol as a reference.


$$\text{\% Inhibition}=\frac{\text{A control }–\text{ A sample}}{\text{A control}}\;\times \;100$$

Very few changes were made to the approach from prior trials (Abd-ElGawad et al., 2020). The exponential curve that showed the relationship between the sample concentration and the amount of DPPH radical still present was used to calculate the inhibitory concentrations. (IC_50_, mg L^−1^) (Parejo et al., 2000).

### Antimicrobial activity and MIC procedure

After 100 µl of the bacterial suspensions were distributed over LB Lauria medium (g/l peptone 10, yeast extract 5, NaCl 0.5, and agar 20). Then, 6 mm-diameter holes were punched into the agar plate. All plates were incubated at 37°C for 18 to 24 h. After incubation, the antibacterial activity was evaluated by measuring the diameter of the inhibitory zone (Ozturk and Ercisli, 2006). The antibacterial activities were tested against four organisms, namely *E. coli* MN1298, *S. aureus* MN2134, *S. epidermidis* MN5130, and *B. subtilis* MN0001. These strains were provided by the Department of Medical Microbiology and Immunology, Faculty of Medicine, Mansoura University, Egypt. The stock solution was prepared by dissolving 0.5 g of dry plant extract in 10 ml of dimethyl sulfoxide. The serial dilutions from the stock solution were made from 40 mg/ml to 5 mg/ml using LB lauria broth medium. Approximately 100 µl of the bacterial suspension were inoculated into the broth medium, and the tubes were incubated at 37°C for 24 h. After incubation, the MIC values were visually determined. The lowest concentration of each extract with no visible growth was recorded as MIC (Eloff, 1998).

### Homology modeling of detoxifying genes using available experimental data

The first step in creating a homology model is to locate a decent template with sufficient sequence identity and an experimental 3D structure that is accessible. The Uniprot database was used to retrieve the desired sequences.

### *In silico* docking studies

Protein DNA gyrase from *E. coli* (PDB ID: 6rks) and undecaprenyl diphosphate synthase (UPPS) (PDB ID: 4H8E) from *S. aureus* were used to stimulate antibacterial activities. The ligands are Limonene PubChem CID 22311 and 13-Octadecenoic acid methyl ester CID 5364506. The structures were obtained from the protein data bank and PubChem. Before the interaction study, the prank website server was used to identify the binding site prediction with potential targets (Qanash et al., 2022). The structures of ligands were minimized in their binding energy using PyRx software (a virtual screening tool). By adding polar hydrogen atoms while removing non-polar hydrogen atoms and water molecules from the molecules, BIOVIA Discovery Studio has generated the proteins. The prepared ligands and protein structures were saved in PDBQT format for calculating energy and autogrid dimension (Hassan et al., 2019).

### Data analysis

The biological data (means standard error) were subjected to a one-way analysis of variance using the computer program Costat (CoHort Software, Monterey, CA, USA) (ANOVA). Duncan’s test was used for comparisons between different treatments. At the p**<**0.05 level, statistical differences were considered significant.

## Results

### Gas chromatography mass spectroscopy

**Figure 1:**
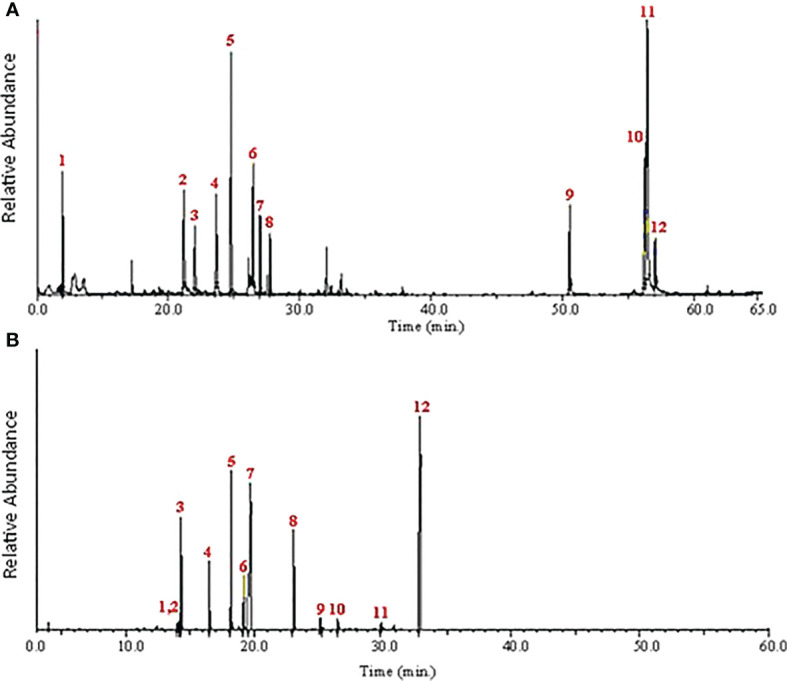
Chromatogram of main components of the hydromethanolic extract of *R. officinalis* and *A. indica* leaves by GC–MS.

This study used GC/MS analysis to examine the hydromethanolic extracts of *R. officinalis* and *A. indica* leaves. The extracted chromatogram listed the chemical components peak areas (%) and retention times as listed in **Figure 1**. A total of 12 components constituting 98.61% of the total MeOH extract of *R. officinalis* leaves were identified during the GC–MS analysis (**Table 2**). Limonene (23.03%) is frequently identified as the main ingredient that was discovered after 24.79 min (**Figure 1A**). Other substances were then classified with higher compositional percentages, such as cis-vaccenic acid (12.91%), *(2E,4E)*-deca-2,4-dienal (11.67%), 2,4,6-trimethyloctane (10.14%), 9,12-octadecadienoic acid (9.77%), *(E)-*non-3-ene (8.65%), and *(E)*-hept-4-enal (7.90%). These substances comprise 61.04% of all the chemicals found in **Table 2**. In contrast, the hydromethanolic extract of *A. indica* leaves is characterized by the high concentration of several physiologically significant chemicals listed in **Table 2**. After 20.14 and 33.13 min, the primary chemical that was discovered was typically recognized as methyl (E)-octadec-13-enoate (23.20%) and (2R)-1,3,8-trimethyl-4-propyl-5-ethyl-2-(1-hydroxyethyl)-7-methoxycardonylethyl-6-γ-methylenecarbonyl-porphyrin (37.40%). The identification of other substances with higher compositional percentages included 4,5-dimethylheptan-3-ol (14.20%), (E)-3,7-dimethylocta-2,6-dienal (11.60%), and methyl docosanoate (7.60%). Of all the compounds discovered, these substances made up 33.40% of the total.

**Table 2:** The extracted leaves of *R. officinalis* and *A. indica* contained the described chemical components.

No.	QID^a^	Component	Rt^b^	Conc. %^c^	Molecular Weight	Molecular formula
		*R. officinalis*				
		Hydrocarbon				
**1**	**2**	(*E*)-non-3-ene	21.20	8.65±0.08	126.24	C_9_H_18_
**2**	**3**	(*E*)-dec-2-enal	22.04	2.96±0.01	154.25	C_10_H_18_O
**3**	**4**	(2*E*,4*E*)-deca-2,4-dienal	23.67	11.67±0.07	152.24	C_10_H_16_O
**4**	**6**	2,4,6-trimethyloctane	26.47	10.14±0.09	156.31	C_11_H_24_
**5**	**7**	(*E*)-undec-2-enal	27.01	2.49±0.01	168.28	C_11_H_20_O
**6**	**8**	(*E*)-hept-4-enal	27.75	7.90±0.03	112.17	C_7_H_12_O
		Fatty acid				
**7**	**10**	9,12-Octadecadienoic acid	56.33	9.77±0.02	280.45	C_18_H_32_O_2_
**8**	**11**	Cis-Vaccenic acid	56.49	12.91±0.04	282.47	C_18_H_34_O_2_
**9**	**12**	Stearic acid	57.07	1.61±0.01	284.48	C_18_H_36_O_2_
		Monoterpene				
**10**	**1**	Eucalyptol	11.97	3.98±0.02	154.25	C_10_H_18_O
**11**	**5**	Limonene	24.79	23.03±0.27	136.24	C_10_H_16_
**12**	**9**	Linalool	50.75	3.50±0.02	154.25	C_10_H_18_O

No.	QID^a^	*A. indica*				
		Hydrocarbon				
**1**	**3**	(*E*)-3,7-dimethylocta-2,6-dienal	14.20	11.60±0.03	152.24	C_10_H_16_O
**2**	**4**	7-methoxy-3,4-dihydro-2*H*-spiro[naphthalene-1,2'-[1,3]dithiolane]	16.52	1.85±0.01	252.39	C_13_H_16_OS_2_
**3**	**5**	4,5-dimethylheptan-3-ol	18.19	14.2±0.03	144.26	C_9_H_20_O
		Lipid (esters of fatty acids)				
**4**	**6**	Methyl oleate	19.16	1.30±0.01	296.50	C_19_H_36_O_2_
**5**	**7**	Methyl (*E*)-octadec-13-enoate	20.14	23.20±0.31	296.50	C_19_H_36_O_2_
**6**	**8**	Methyl docosanoate	25.33	7.60±0.02	354.62	C_23_H_46_O_2_
**7**	**11**	*bis*(2-ethylhexyl) adipate	30.20	0.90±0.01	370.57	C_22_H_42_O_4_
		Alkaloid				
**8**	**9**	3-(*tert*-butyl)-4,5,6,7-tetrahydro-1*H*-indole	25.50	0.80±0.05	177.29	C_12_H_19_N
**9**	**10**	6-methyl-9H-carbazole-3,4-diyl)*bis*((4-chlorophenyl)methanone	26.54	0.30±0.00	458.34	C_27_H_17_Cl_2_NO_2_
**10**	**12**	(2*R*)-1,3,8-trimethyl-4-propyl-5-ethyl-2-(1-hydroxyethyl)-7-methoxycardonylethyl-6-γ-methylenecarbonyl-porphyrin	33.13	37.40±0.41	690.89	C_42_H_50_N_4_O_5_
		Sesquiterpene				
**11**	**1**	Caryophyllene oxide	13.90	1.60±0.01	220.36	C_15_H_24_O
		Monoterpene				
**12**	**2**	Citral	14.05	0.50±0.00	152.24	C_10_H_16_O

^a^ Quantitative ID, ^b^ retention time (min), ^c^ average concentration of three replications ± standard deviation.

**Figure 2:**
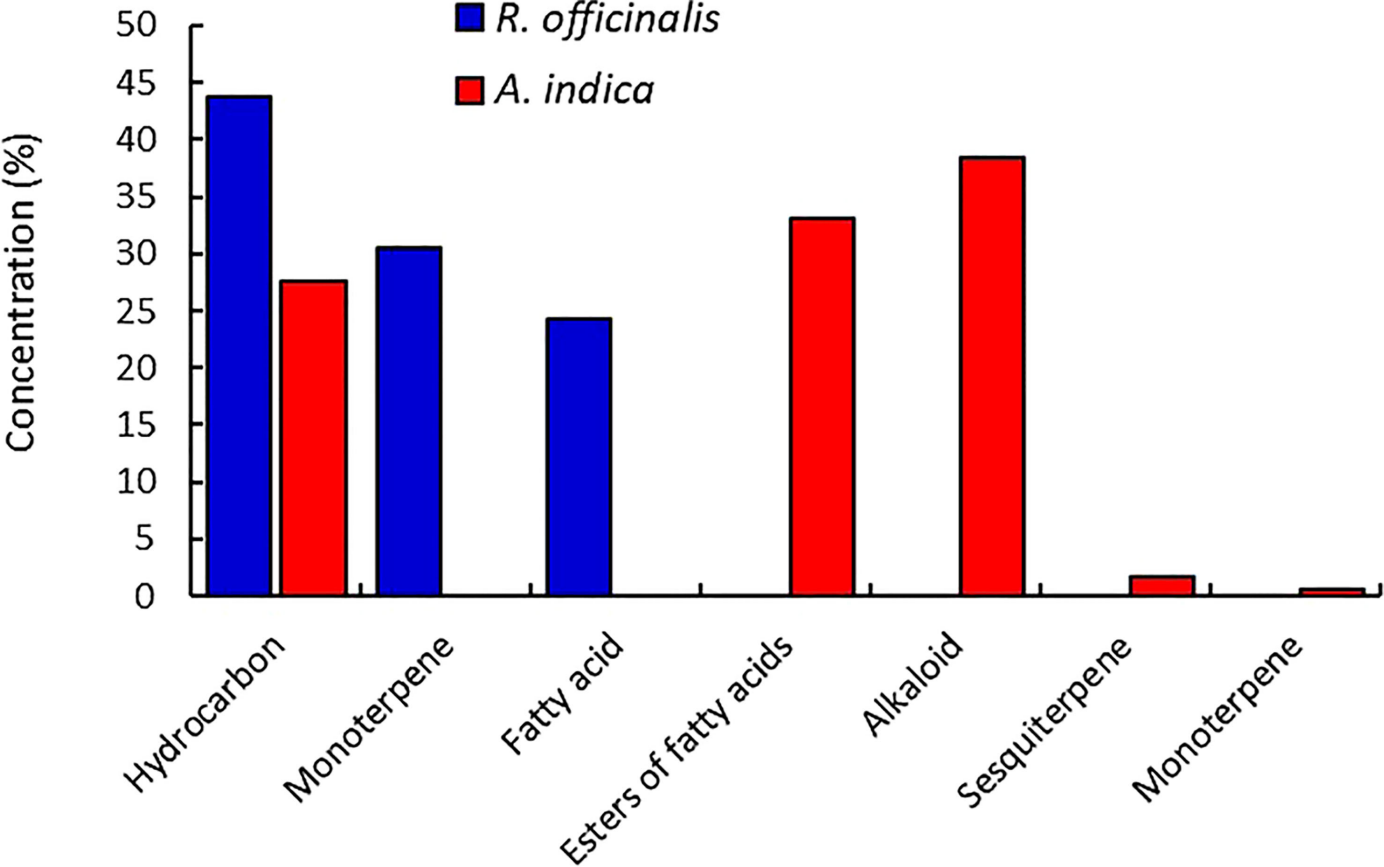
By using GC–MS analysis, the classified chemical components from the extracted leaves of *R. officinalis* and *A. indica* were discovered.

In a MeOH extract of *R. officinalis* leaves, the components of monoterpene were recorded with retention times ranging from 11.97 to 50.75 min, and the components of fatty acids were recorded at low relative abundances between 56.33 and 57.07. Retention times between 21.20 and 27.75 min were used to determine hydrocarbons as the most prevalent constituents (**Figure 2**). While in the MeOH extract of *A. indica*, the low relative abundances of monoterpene and sesquiterpene components were observed at 14.05 and 13.90 min, respectively. Retention times between 14.20 and 18.19 min were used to pinpoint hydrocarbons as the most prevalent constituents. However, the retention times for the constituents of fatty acid esters were 19.16 and 30.20 min. The components of the alkaloids were found in high relative abundances at 20.25 and 33.13 min (**Figure 2**).

### Biological properties of the plant extracts

#### Toxicity bioassay

##### Mortality and repellence activities

In this study, both extracts of *R. officinalis* and *A. indica* exhibited different degrees of toxicity against *T. castaneum*. The estimated LC_50_ and LC_90_ values of *R. officinalis* against *T. castaneum* were 1.470 and 0.159 mg/ml, whereas the calculated LC_50_ and LC_90_ values of *A. indica* against *T. castaneum* were 2.588 and 0.345 mg/ml at 6 and 72 h, respectively (**Table 2**). Our findings are consistent with several studies that found *R. officinalis* and *A. indica* to be powerful deterrents against the main pest species in stored grain systems (Ahmadi et al., 2008). According to Khalil et al. (2015), *T. castaneum* was susceptible to *R. officinalis* at LC_50_ and LC_90_ values of 0.18 and 0.33 mg/ml at 0.2 mg/ml after 24 h. Additionally, Asifa et al. (2012) showed that the ethanol extract of *A. indica* had an LC50 value of 6.27 and 0.58 mg/ml after 24 and 72 h, respectively.

The lesser grain borer (*Rhyzopertha dominica*), the rice weevil (*Sitophilus oryzae*), the granary weevil (*S. granaries*) and the red flour beetle (*T. castaneum*), are the most frequent insects that attack dried grains and pulses in many countries, including Egypt, India, Pakistan, Mexico, and others (Rajashekar et al., 2012; Abdelsalam et al., 2019). In the present study, a mortality bioassay was conducted to check out the toxicity efficacy of the hydromethanolic extracts obtained from *R. officinalis* and *A. indica* leaves on the third instar *T. castanum* larva, as shown in **Table 2**. Overall, the mortality rate in the *T. castanum* larval stage increased as the concentration and time exposure to the investigated extracts increased. Among which the *A. indica* extract showed potent fumigant activity against the third instar larval stage of *T. castanum* pest. The hydromethanolic extracts (0.4 mg/ml) from *A. indica* leaves exhibited the highest mortality rate of 100% and 98.4% against the *T. castanum* larva after 72 h and 48 h of exposure time, respectively, and a good toxicity effect of 92.1% against the *T. castanum* larva was observed at a 0.4 mg/ml concentration of *R. officinalis* leaves after 72 h. While after 12 h and 6 h of exposure time, *R. officinalis* and *A. indica* extracts had a minimal mortality (5.3% and 18.6%) impact on *T. castanum* larvae, respectively (**Table 3**). The botanical oils of *Simmondsia chinensis* and *R. officinalis* plants grown in Egypt demonstrated substantial activity against *T. castaneum* adults, providing 95% and 67% mortality, respectively, at 3 DAT, according to Shawer et al. (2022). Our data support their findings. At a concentration of 27.76 L/L after 72 h, *R. officinalis* oil extract demonstrated an 83.3% death rate against *T. castaneum* (Khoobdel et al., 2017). On the other hand, Nadeem et al. (2012) discovered that the ethanol extract of *A. indica* showed larvicidal efficacy against *T. castaneum* with 64.44% at 100 mg/ml after 72 h. The effectiveness of *A. indica* methanol extracts against *T. castaneum* larvae with a concentration of 37.52% after 24 h was also examined by Sami et al. (2016).

**Table 3:** *T. castenum*, 3rd instar larval mortality (%) from using different concentrations (mg/ml) of hydromethanolic extracts from *R. officinalis* and *A. indica* leaves.

Treatment (70% MeOH)	Dose Conc. (mg/ml)	Mean % of mortality (mean ± SE)				
		6h	12h	24h	48h	72h
	Control	0.00	0.00	0.00	0.00	1.0 ± 0.2
***R. officinalis***	0.1	0.00	5.3 ± 0.21 ^e^	17.9 ± 1.8 ^e^	35.6 ± 4.8 ^d^	47.5 ± 4.2 ^c^
	0.2	0.00	17.3 ± 1.0 ^e^	34.6 ± 5.6 ^d^	46.0 ± 2.6 ^c^	72.9 ± 5.4 ^b^
	0.3	9.4 ± 1.57 ^e^	30.5 ± 3.1 ^d^	39.4 ± 2.7 ^d^	55.4 ± 3.7 ^c^	80.6 ± 6.7 ^a^
	0.4	13.2 ± 5.0 ^e^	29.7 ± 1.8 ^d^	50.6 ± 4.9 ^c^	79.4 ± 6.2 ^b^	92.1 ± 7.2 ^a^
	LC_50_	1.470	0.595	0.375	0.238	0.159
	LC_90_	2.588	1.068	0.701	0.462	0.345
***A. indica***	0.1	18.6 ± 3.4 ^e^	32.5 ± 2.9 ^d^	38.9 ± 2.7	55.3 ± 4.5 ^c^	70.3 ± 3.7 ^b^
	0.2	22.5 ± 2.2 ^d^	31.5 ± 2.4 ^d^	43.7 ± 3.4 ^c^	66.2 ± 5.1 ^b^	91.4 ± 4.6 ^a^
	0.3	35.2 ± 1.9 ^d^	38.1 ± 1.8 ^d^	59.3 ± 5.8 ^c^	90.2 ± 7.6 ^a^	96.7 ± 5.5
	0.4	47.4 ± 2.4 ^c^	59.6 ± 4.1 ^c^	86.7 ± 7.3 ^a^	98.4 ± 6.4 ^a^	100 ± 6.8^a^
	LC_50_	0.427	0.342	0.222	0.148	0.102
	LC_90_	0.786	0.662	0.428	0.321	0.281

Within a column, there is a significant difference between data with different small letters (P< 0.05). LC50 stands for the median extract concentration at which 50% death, and LC90 for the median extract concentration at which 90% death.

In the present results, *T. castenum’s* 3rd instar larval repellant percentage increased as the concentration and time exposure of methanolic extracts from *R. officinalis* and *A. indica* leaves increased. The obtained results in **Table 4** indicate that the biopesticides showed excellent repellency against *T. castaneum* larvae and that there was a significant difference in all treatments of selected plant materials. Among these, the *A. indica* extract demonstrated the highest levels of repellent activity (81.4% and 93.4%) against *T. castenum* larvae at 0.2 mg/ml concentration after 4 and 5 h of exposure, respectively. After 4 and 5 h of treatments with 0.2 mg/ml of *R. officinalis* extract, good repellent rates of 64.9% and 80.7% were seen. In the current study, *R. officinalis* and *A. indica* extracts effectively repelled *T. castaneum*. This supports the results of numerous studies using *R. officinalis* and *A. indica* as insect repellents for stored goods storage (Islam and Talukder, 2005). However, certain adverse results of the extracts against *T. castaneum* may be connected to the plant species used, the extraction solvent, the species of accessible vector, and other substances (phenols, flavonoids, alkaloids, etc.). The gene expression results represented in **Table 5** showed that the gene toxicity in *T. castenum* larvae under selected plant extract treatment was demonstrated by induction. The relative expression level of the CYP 450 gene was 5.94- and 5.56-fold changes in mRNA in *T. castenum* larvae exposed to 5 ppm malathion for 2 h and 1 h, respectively, followed by 2.36-fold changes in mRNA in *T. castenum* larvae exposed to *A. indica* leaf extract after 1 h vs the reference gene (housekeeping gene, β-Actin). Real-time analysis results in **Table 5** showed that the gene expression profile in *T. castenum* larvae under selected plant extract treatment was demonstrated by induction; the relative expression level of the CYP sim gene was 5.94 and 5.56-fold changes in mRNA in *T. castenum* larvae exposed to 5 ppm malathion for 2 h and 1 h, respectively, followed by 4.74-fold changes in mRNA in *T. castenum* larvae exposed to *A. indica* leaf extract after 2 h as compared with the reference gene (housekeeping gene, β-Actin).

**Table 4:** *T. castenum*, 3rd instar larval repellence (%) from using different concentrations (mg/cm^2^) of hydromethanolic extracts from *R. officinalis* and *A. indica* leaves.

Treatment (70% MeOH)	Dose Conc. (mg/ml)	Mean % of mortality (mean ± SE)				
		1h	2h	3h	4h	5h
	Control	0.0±0.0	0.0 ± 0.0	0.0 ± 0.0	0.0 ± 0.0	1.0 ± 0.32
***R. officinalis***	0.05	8.2 ± 2.0 ^e^	18.4 ± 1.9 ^e^	19.7 ± 2.0 ^e^	27.3 ± 2.1 ^d^	43.1 ± 3.4 ^c^
	0.10	17.3 ± 1.2 ^e^	21.6 ± 0.9 ^d^	25.4 ± 1.8 ^d^	33.4 ± 2.4 ^d^	49.7 ± 4.2 ^c^
	0.15	18.6 ± 0.9 ^e^	38.4 ± 3.1 ^d^	46.2 ± 3.1 ^c^	42.7 ± 4.0 ^c^	71.0 ± 5.1 ^b^
	0.20	24.6 ± 2.0 ^d^	30.4 ± 2.1 ^d^	41.3 ± 1.8 ^c^	64.9 ± 2.6 ^b^	80.7 ± 6.0 ^a^
***A. indica***	0.05	16.4 ± 0.7 ^e^	26.4 ± 1.8 ^d^	31.4 ± 2.0	37.9 ± 2.1	40.2 ± 3.1 ^c^
	0.10	18.6 ± 0.9 ^e^	27.3 ± 2.4 ^d^	25.8 ± 2.6 ^e^	41.2 ± 2.7 ^c^	64.4 ± 3.7 ^b^
	0.15	27.3 ± 1.1 ^d^	37.5 ± 1.7 ^d^	52.3 ± 3.4 ^c^	66.7 ± 4.5 ^b^	79.4 ± 5.1 ^b^
	0.20	39.3 ± 2.0 ^d^	51.3 ± 4.8 ^c^	69.3 ± 5.4 ^b^	81.4 ± 6.0 ^a^	93.4 ± 4.9 ^a^
**Malathion**	5 ppm	35.4 ± 2.8 ^d^	50.3 ± 4.6 ^c^	73.4 ± 6.2 ^b^	68.4 ± 5.7 ^b^	84.1 ± 6.1 ^a^

**Table 5:** Relative gene expression in *R. officinalis* and *A. indica* leaves.

Treatment	Dose (mg/ml)	CYP 4Q7 gene		CYP 450		CYP sim gene	
		After 1h	After 2h	After 1h	After 2h	After 1h	After 2h
70% methanol	(control)	1.23	1.14	1.23	1.14	1.23	1.14
*R. officinalis*	6.0	5.12	5.24	1.8	1.94	2.33	2.40
*A. indica*	6.0	8.14	9.05	2.36	2.09	4.41	4.74
Malathion	5ppm	5.56	5.94	5.56	5.94	5.56	5.94

### Protein–ligand interaction of the detoxification genes

**Figure 3:**
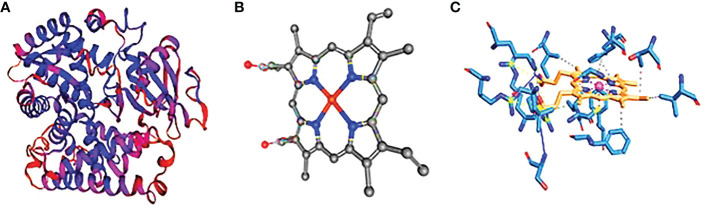
Cytochrome P450. **(A)** The three-dimensional structure of cytochrome P450 showing the hydrophobic and hydrophilic groups, **(B)** the ligand structure, and **(C)** the protein–ligand interaction showing the hydrophobic bonds.

The protein cytochrome P450 (CYP4B1) is predicted to have a molecular weight of 379 amino acids and a theoretical pI of 8.77. There are 13 negatively charged residues (Asp + Glu) and a total of 17 positively charged residues. **Figure 3A** contains 6,117 atoms in total. The protein is categorized as stable, with a calculated instability index (II) of 39.43. The interaction of the protein–ligand with HEM (protoporphyrin IX) as a ligand. Based on our data, hydrophobic connections, totaling 10 contacts between carbon and hydrogen atoms, are by far the most frequent interactions in protein–ligand complexes. The labeled residues are 118A, 137A, 302A, 305A, 309A, 313A, 370A, 373A, 435A, and 443A. The distance between interacting carbon atoms is 3.63, 3.34, 3.97, 3.55, 3.50, 3.43, 3.70, 3.84, 3.51, and 3.41A (**Figure 3B**). Unexpectedly, alanine was the second most common donor and the third most common acceptor of hydrogen bonds. This is probably because it does not have a sidechain to cover the backbone atoms and has a more flexible backbone to fit the confines of hydrogen bonding better. The model with the 437A residue exhibits hydrogen bonding as the second most common type of interaction, with a distance of 3.81 A° between the donor and the receptor. Among the interactions are the salt bridges between 105A, 130A, 375A, and 440A° residues. Due to the presence of six nitrogen atoms in the side chains, arginine forms more hydrogen bonds than glycine. We discovered that the median lengths between heavy atom bonds were all roughly 4.0. Additionally, the median separations between charged and neutral hydrogen bonds were nearly equal at 1A°.

Cytochrome CYP4Q7 has 543 amino acids and a calculated pI of 9.18. 50 positively charged residues (Asp + Glu) and 100 negatively charged residues make up the total (Arg + Lys) of 59, with a total number of atoms of 8,552. The calculated instability index (II) of 37.69 categorized the protein as stable. The interaction between a positively charged nitrogen and a negatively charged oxygen was the most frequent form in our study (also known as a salt bridge) (3,111 interactions). Because the favorable binding energy that results from forming a salt bridge is insufficient to compensate for the energetic cost of dissolving charged groups, salt bridges have little effect on the stability of proteins. The interacting atoms are at distances of 3.96, 4.67, 4.87, 3.68, and 3.39 A°. They are 79A, 107A, 363A, and 430A°. **Figure 3C** shows the full structure of CYP Sim, which has 532 amino acids and a calculated pI of 9.43. 52 positively charged residues and 62 negatively charged amino acids make up the total. Hydrophobic hydrogen bonds, water, and salt bridges illustrate the interaction between proteins and ligands. The hydrophobic bonds between these residues are: 104A, 105A, 123A, 290A, 293A, 357A, 358A, 424A, and 436A, with distances of 3.50, 3.87, 3.60, 3.80, 3,73, 3.23, 3.74, 3.92, 3.66, and 3.88 A°. The hydrogen bonds between 92A and 93A have a distance of 1.50 A°. The salt bridges between 88A, 116A, and 429A have distances of 3.19, 4.56, and 4.27 A°. The water bridges between 88A and 425A have distances of 2.65 and 2.61 A°.

### Antioxidant activity—DPPH assay

The ability of the hydromethanolic extract to scavenge DPPH free radicals was used to measure the antioxidant activity of the substance at various doses (5–50 mg/L). It was clearly shown that the scavenging action was concentration dependent. The inhibition percentage of MeOH extracts was found to be 75.06% at 50 mg/L (**Table 6**). Using half-maximum inhibitory concentration (IC50) values, the results show how plant extracts and the standard affect the DPPH radical. A decreased IC50 value shows an increased ability to scavenge DPPH radicals. The results shown in **Table 6** support the hypothesis that the resin extract had the highest antioxidant scavenging activity with an IC50 of 26.86 mg/L. The highest DPPH radical scavenging activity of MeOH extract appears to be due to the high concentration of sesquiterpenoids, diterpenes, triterpenes, and sterols in myrrha extracts, which may act as electron donors and react with free radicals to transform them into more stable products and prevent radical chain reactions.

**Table 6:** Radical scavenging activity (%) and IC_50_ values (mg/L) were determined using the DPPH test with methanol-extracted *R. officinalis* and *A. indica* and standard ascorbic acid at varied doses.

Conc. (mg/L)	Radical Scavenging Activity (%)	
	*R. officinalis*	*A. indica*
5	3.12 ± 0.10^F^	7.71 ± 0.26^E^
10	18.49 ± 0.62^E^	23.08 ± 0.77^D^
20	37.01 ± 1.23^D^	41.60 ± 1.39^C^
30	46.27 ± 1.54^C^	50.86 ± 1.95^B^
40	53.04 ± 2.26^B^	57.63 ± 2.68^B^
50	67.76 ± 2.90^A^	72.35 ± 3.24^A^
IC_50_ (mg/L)	35.83	28.68
LSD_0.05_	2.61***	1.85***
	Ascorbic acid	
1	3.21 ± 0.01^F^	
2.5	10.38 ± 0.03^E^	
5	37.29 ± 0.19^D^	
10	47.07 ± 0.51^C^	
15	62.94 ± 1.42^B^	
20	71.63 ± 1.55^A^	
IC_50_ (mg/L)	12.34	
LSD_0.05_	1.28***	

Standard deviation average (n = 3) values. As each test was conducted on those two means, LSD_0.05_ expressed the predicted least of the smallest significance between those two means (calculated by Factorial ANOVA). ***, significant at P≤ 0.001.

### Antimicrobial activity

To investigate the antibacterial activity of plants against pathogenic bacteria, plant extracts were tested against four bacterial strains, but only two strains, *E. coli* MN1298 and *S. aureus* MN2134, showed the most potent results. The disc diffusion method was used to evaluate and determine the susceptibility of microorganisms. **Table 7** lists the antibacterial qualities of plant extracts. At a dosage of 20 mg/ml, extracts of *R. officinalis* and *A. indica* leaves were both discovered to be the most efficient extracts against gram-positive (*S. aureus*) and gram-negative (*E. coli*) bacteria. The MIC values of the effective plant extracts were calculated using the disc diffusion method, and the results were used to determine the bacteriostatic and bactericidal properties of the plant extracts. The amount of plant extract shown to be effective is shown in the table below (**Table 7**). At a dosage of 20 mg/ml, the extracts were shown to have an inhibitory effect, with the zones of inhibition for *S. aureus* and *E. coli* measuring 6.5 mm, 10.5 mm, 12.21 mm, and 15.00 mm, respectively. The findings are in line with those made public by Jeevalatha et al. (2022), who found that natural antibacterial methyl ester was effective even at low concentrations. These compounds generally inhibit Gram-negative bacteria in lower quantities than gram-positive bacteria.

**Table 7:** MIC of methanolic plant extract (20 mg/mL) against several bacterial strains and antimicrobial screening test.

Treatments	Inhibition zone (mm)		
	*E. coli*	*S. aureus*	
*R. officinalis*	11.0	9.0	
*A. indica*	5.0	11.0	

MIC			
Treatments	Concentration (mg/ml)	Inhibition zone (mm)	
		*E. coli*	*S. aureus*
*R. officinalis*	5	–	–
	10	–	–
	15	–	–
	20	–	–
	25	++	++
*A. indica*	5	–	–
	10	–	–
	15	–	–
	20	–	–
	25	++	++

Positive (+), growth; Negative (–), absence of growth.

### Docking studies

**Figure 4:**
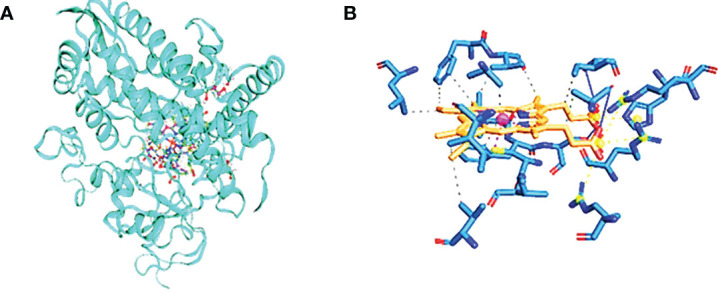
Cytochrome CYP4Q7. **(A)** The three-dimensional structure of cytochrome, showing CYP4Q7 the hydrophobic and hydrophilic groups, and **(B)** the protein–ligand interaction showing the salt bridge bonds.

**Figure 5:**
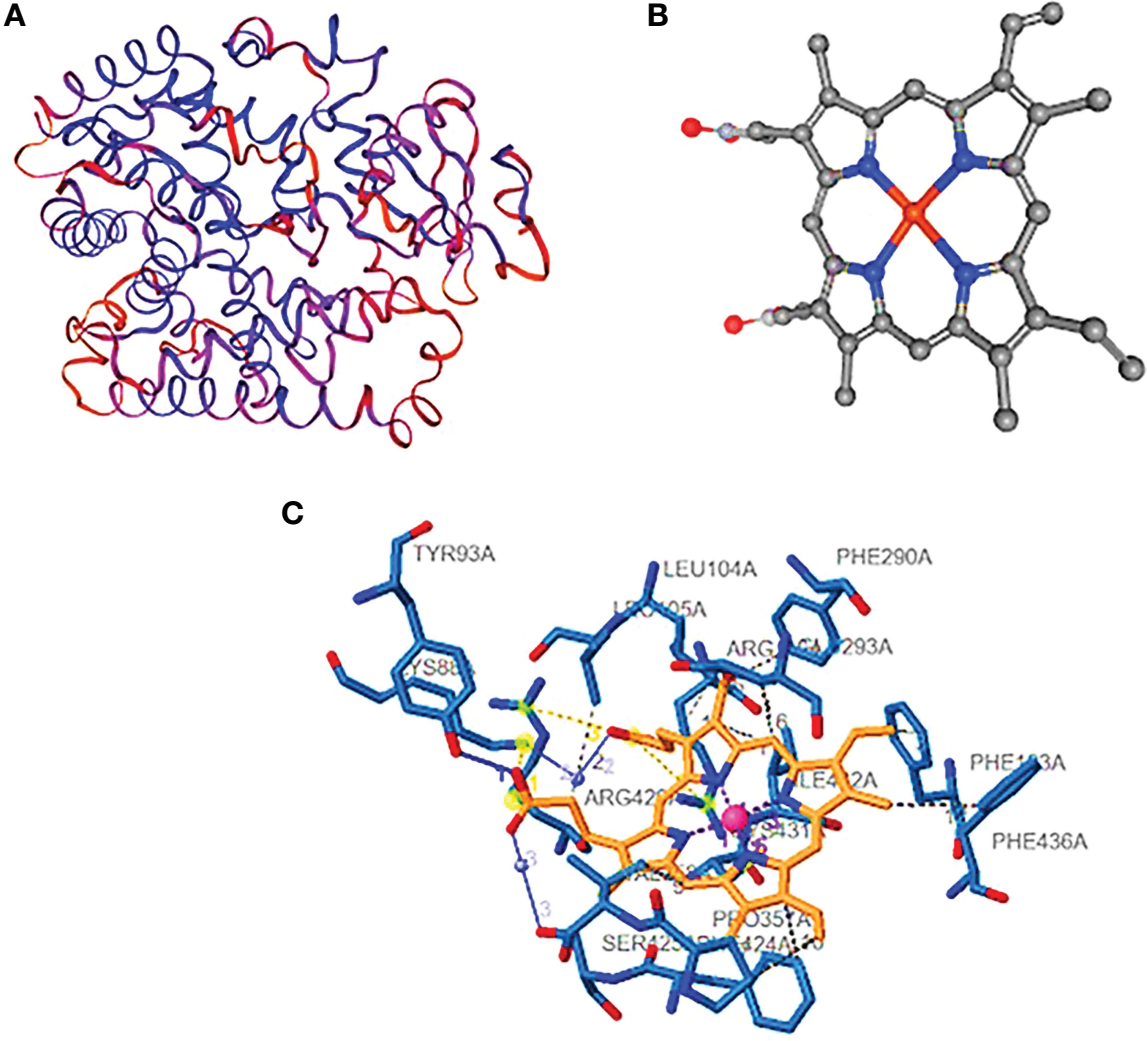
Cytochrome P sim. **(A)** The predicted three-dimensional structure of CYP sim, **(B)** the ligand structure, and **(C)** the protein–ligand structure with the hydrogen bonds.

**Figure 6:**
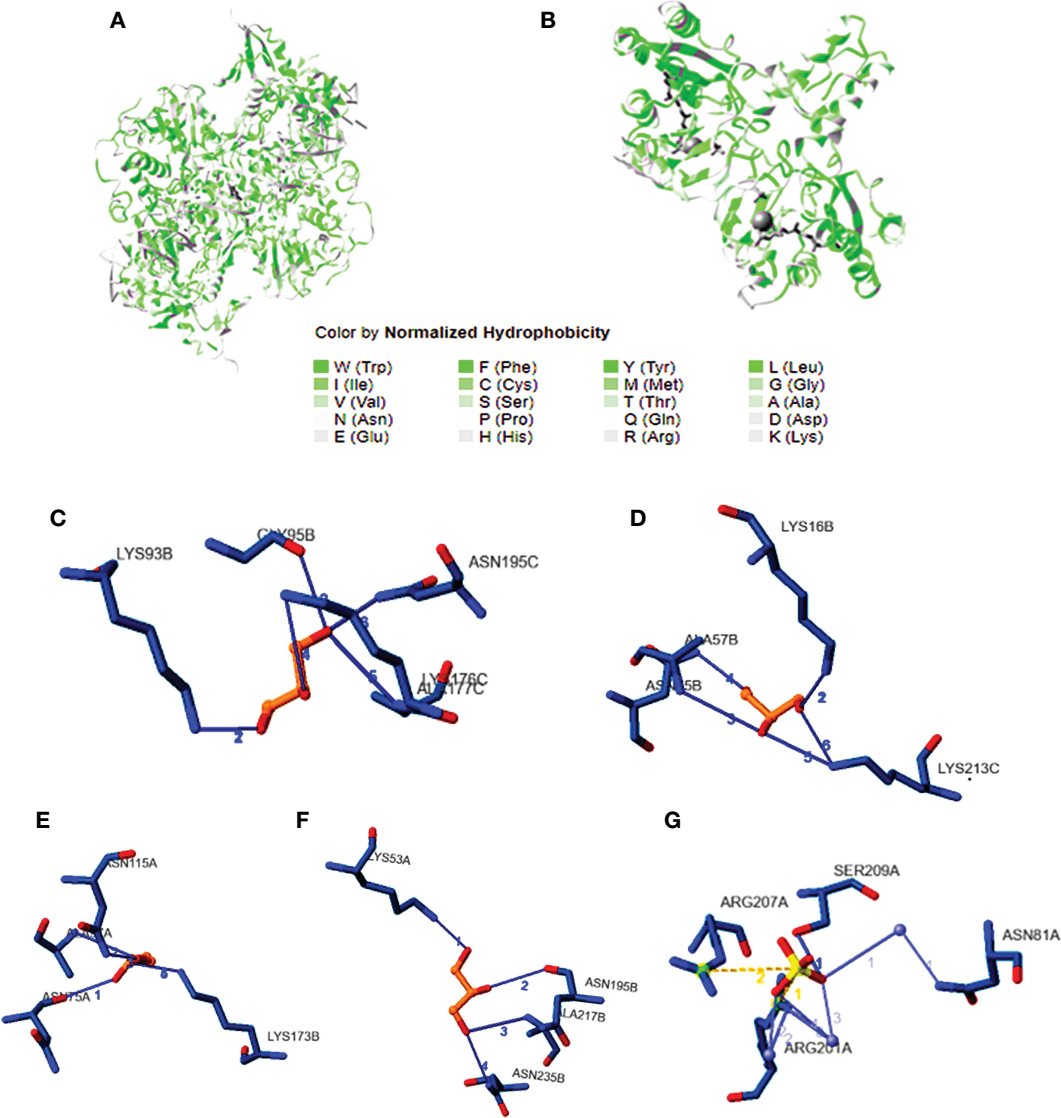
The three-dimensional structure of the selected docked proteins with the interacting ligands attached at different interacting chains.

**Figure 7:**
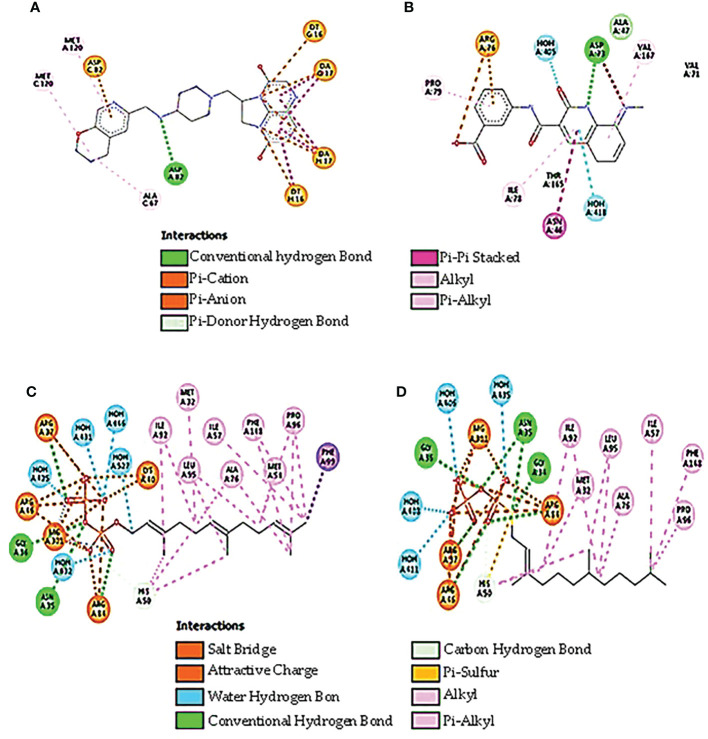
The best docking confirmation of ligands with the selected proteins. **(A)** Limonin with DNA gyrase, **(B)** methyl ester with DNA gyrase, **(C)** limonin with UPP, and **(D)** methyl ester with UPP.

DNA gyrase anticipated molecular weight A subunit (6rks) having a mass of 394.36 kDa, a total of 12,572 atoms, and two different protein chains. There are 17 negatively charged residues (Asp + Glu) and a total of 22 positively charged residues (Arg + Lys). A total of 8,222 atoms makes up the entire system (**Figure 4A**). The instability index (II) is computed to be 44.43; this classifies the protein as stable. The ligands are small molecules of glycerol attached between chains A and B by hydrogen bonds at residues 75A, 95A, 115A, and 173B with distance ranges from 2.55 to 2.89 A°. The second interacting chains are B and C attached with hydrogen bonds at 16B, 57B, 75B, and 213C with a distance of 2.98 to 3.14 A°. The third interacting chains are B and C with residues 93B, 95B, 176C, 177C, and 195C. The fourth molecules are interacting with hydrogen bonds between residues 53A, 195B, 217B, and 235B. The predicted total weight of undecaprenyl diphosphate synthase is 30 kDa with only one unique protein chain. The protein–ligand interaction is illustrated in **Figure 4B**. The molecules are attached by hydrogen bonds at residue 209A with a distance of 2.48 A°. All the extracted compounds were docked into the active site of DNA gyrase A subunit and undecaprenyl diphosphate synthase (4H8E) for *S. aureus* and then were analyzed for binding free energy and their interactions with proteins (**Figure 5**). **Table 8** summarizes the binding free energy in Kcal/mole and interactions between the most concentrated compounds and the selected proteins with their minimization energy. Compound limonene, 13-octadecenoic acid, and methyl ester showed good affinity against the selected proteins. The area containing the active site was filled with limonene. Limonene and DNA gyrase have a binding affinity of −7.85kcal/mol. Studies involving flavonoids were found to have a lower binding affinity for the DNA gyrase than those with limonene, which acts as an inhibitor (**Figure 6**). Mohammed et al. (2015) found Daidzein (+2.60 kcal/mol), Ex-emestane (−3.96 kcal/mol), Kaempherol (−3.75 kcal/mol), Letrozole (−3.55 kcal/mol), Rutin (−1.11 kcal/mol), Quercetin (−1.78e+32 kcal/mol), and Orlistat (−4.86 kcal/mol) all have DNA gyrase protein receptor binding energies. The first ligand showed nonpolar interaction within *E. coli* at chain A between Ile 8, Thr 9, Pro 10, Val 11, and Asn 12. The dimensions of the docked grid box were x = 158.404, y = 158.386, and z = 148.093A, with hydrophobicity ranging from −0.8 to −1.4. In the case of ligand 2, the hydrophobicity ranges from −0.4 to −1.2 within Asp 251, His 252, Arg 256, Glu 257, and Thr 258. The dimension of the grid box was x = 25.117, y = 22.494, and z = 17.1949. This ligand showed conventional hydrogen bonds within *S. aureus* Asp (A 82), alky and pi-alkyl bonds between Ala (C 67), Met (C 120), and Met (A 120), pi anion, pi cation bonds between DA (G17), DT (G 16), DA (H 17), and DT (H 16). The pocket atoms are ILE (C74), Gly (C71), VAL (A70), GLY (A71), ILE (A74), and ASP (C 82). The minimization energies of the selected three compounds were 9.958, 12.464, and 6.874 (Kcal/mole), with acceptable affinity for the pocket site (**Figure 7**).

**Table 8:** The Binding energies against the extracted metabolites.

Ligand	Binding Affinity (Kcal/mole)	rmsd/upper binding	rmsd/lower binding
4HSE_CID 5364506	**-5**	20.606	18.005
4H8E_CID 5364506	-4.9	2.496	1.558
4H8E_CID 5364506	-4.9	20.019	17.532
4H8E_CID 5364506	-4.8	2.119	1.233
6rks_CID5364506	-4.8	20.334	**17.351**
6rks_CID 5364506	-4.7	6.397	3.742
6rks_CID 5364506	-4.6	2.526	1.476
6rks_CID 5364506	-4.6	4.758	3.592

Ligand	Binding Affinity	rmsd/ub	rmsd/lb
6rks_CID 22311	-4.2	6.262	4.291
6rks_CID 22311	-4.2	29.631	27.568
6rks_CID 22311	-4.2	6.585	3.838
6rks_CID 22311	-4.1	4.849	2.72
4H8E_CID 223 11	-4.1	5.151	2.951
4HSE_CID 22311	**-4.l**	6.502	4.199
4H8E_CID 223 11	-4.1	4.242	2.208
4H8E_CID 223 11	-4	4.544	2.923

## Discussion

It is well known that the findings of the current experiment and the chemical profiles of the previously analyzed *R. officinalis* and *A. indica* leaf extracts differ in terms of the diversity or quantity of the components. However, some reports in the literature by Hameed et al. (2015) suggest that eucalyptol and cis-vaccenic acid represent the higher compositional percentages of the methanolic seed extract of Iraqi *R. officinalis*. The present result, however, shows that the extract does not include α-pinene, camphene, neocurdione, galanthamine, or retinoic acid. The primary constituents of the essential oil of Indian *R. officinalis* leaf according to Jeevalatha et al. (2022) were α-pinene (13.64), 1,8-Cineole (41.75%), isopulegol (13.66%), limonene (5%), eucalyptol (6.71%), and linalool (1.19%). Additionally, n-octane, limonene, and linalool. 2,4,6-Trimethyloctane was found in the essential oils of *R. officinalis* leaf extract from six different geographical locations: Yemen, the Western Cape, Kenya, Alabama, and the Western Cape of South Africa (Satyal et al., 2017). According to Elbanna et al. (2018), the primary saturated fatty acids in the KSA eco-species of *R. officinalis* leaf extract were stearic acid (5.96%), palmitic acid (8.9%), linoleic acid (5.96%), and oleic acid (41.2%). In addition to genetic influences, various environmental conditions may also contribute to the accumulation of active secondary metabolites in the wild plant during its developmental and growth phases (Liu et al., 2015; Abd-ElGawad et al., 2020). Recent investigations have also demonstrated that the volatile chemicals (E)-3,7-dimethylocta-2,6-dienal are the major constituents in the fruit of *Litsea pungens* and the leaves of *Melaleuca leucadendron* and *Eucalyptus staigeriana* (Paula et al., 2020; Pu et al., 2021). According to Choppa et al. (2015) and Macedo et al. (2021), the main component of *Garcinia cambogia* seed oil from Bureau and in the genus *Triplaris* was methyl (E)-octadec-13-enoate. Caryophyllene oxide is a naturally occurring sesquiterpene that is present in a variety of the extracted essential oils, including *Syzygium aromaticum*, *Piper nigrum*, and *Cannabis sativa*, among others, in respectable amounts (Ghelardini et al., 2001; Ormeño et al., 2008). The results showed that the effectiveness of phytochemicals against mosquito larvae may vary greatly depending on the plant species, plant parts employed, age of plant parts (young, mature, or senescent), extraction solvent, and accessible vector species (Anupam et al., 2012). Additionally, citral is a significant ingredient in essential oils produced from a variety of herbal plants, including the leaves of *Cymbopogon flexuosus* and *Verbena officinalis*. Citral is also utilized as a food additive and as a scent ingredient in cosmetics (Nakamura et al., 2003; Chaouki et al., 2009). Diverse extracts from various plants have distinctive botanical and medical benefits that, when used properly, may not have negative consequences for the environment, human and animal health, or either (Bossou et al., 2015). When plant parts, EOs, extracts, or powders are added with grains, post-embryonic development, reproduction inhibition, induction of mortality of insect eggs, and progeny generation of stored product insects have all been observed to be reduced (Mackled et al., 2019). Chemical control tools are the main tactics for eradicating these pests from stored goods. However, with the widespread use of synthetic pesticides, there are serious concerns regarding insect resistance, residues on cereals, and increased unfavorable environmental effects. Finding eco-friendly methods is therefore urgently needed (Shawer et al., 2018).

Earlier studies by Fraternale et al. (2011) corroborate this. According to a comparison of their oils, myrrha resin extracts in MeOH and hexane showed the highest levels of DPPH radical scavenging activity, according to Mohamed et al. (2014). The same researchers attributed this outcome to three newly identified furano-sesquiterpenoids that were extracted from MeOH and hexane myrrha extracts and have the greatest capacity to scavenge DPPH radicals: myrhone, 3-methoxy-furanogermacradien-6-one, and 2-methoxy-furanogermacren-6-one. The antioxidant capacity of bioactive compounds is commonly determined by reactive oxygen species, such as phenolics, fatty acids, terpenes, oxygenated hydrocarbons, or carbohydrates that can scavenge or stabilize free radicals (Salama et al., 2022). In a different study, the resin essential oil had greater antioxidant activity at the same dose than the resin’s methanol extract did in terms of scavenging DPPH radicals (Al-Harrasi et al., 2013).

This study tested and analyzed the antibacterial efficiency of limonene against *S. aureus*. According to our research, the lowest concentration of limonene needed to inhibit *S. aureus* from growing and reproducing is 20 ml/L. The major factor in the ability of limonene to perform an antibacterial activity was the degradation of cell shape and structure (Satyal et al., 2017). *E. coli* was significantly inhibited by methyl ester compounds, which could do so through the following mechanisms: First, their potential to impair cell membranes’ and walls’ permeability and integrity even causes cell death. This conclusion may be supported by the leakage of proteins, nucleic acids, and ATPase (Satyal et al., 2017). Additionally, by interfering with the TCA and glycolysis enzyme system functions and blocking ATPase, *E. coli* bacterial respiration and energy metabolism were damaged. This was done to prevent the creation and breakdown of ATP.

## Conclusion

In conclusion, twelve components from the MeOH extract of each *R. officinalis* and *A. indica* leaf were identified by GC–MS analysis. In *R. officinalis* extract, limonene is typically positioned as the main component (23.03%); however, other chemicals were later categorized with larger compositional percentages. These substances represented 61.04% of the total discovered compounds, including cis-vaccenic acid, (2E,4E)-deca-2,4-dienal, 2,4,6-trimethyloctane, 9,12-octadecadienoic acid, (E)-non-3-ene, and (E)-hept-4-enal. While the main chemicals identified in the *A. indica* extract were methyl (E)-octadec-13-enoate (23.20%) and (2R)-1,3,8-trimethyl-4-propyl-5-ethyl-2-(1-hydroxyethyl)-7-methoxycardonylethyl-6-methylenecarbonyl-porphyrin (23.03%), other substances with higher compositional percentages included 4,5-dimethylheptan-3-ol, (E)-3,7-dimethylocta-2,6-dienal, and methyl docosanoate. These substances represent 33.40% of the total. The leaves of *R. officinalis* and *A. indica* showed beneficial biological traits such as larvicidal efficacy against *T. castenum* larvae as well as antioxidant and antibacterial activities. The MeOH extract of the plant samples also more effectively killed *T. castaneum* larvae, with the extracts of *A. indica* and *R. officinalis* having the highest levels of repellent action (93.4% and 80.70%, respectively) after 5 h of treatment at a concentration of 0.2 mg/ml each and the highest levels of lethal concentrations (LC50: d 2.588 mg/ml, and LC90: values of 0.159 and 0.345 mg/ml at 6). The leaf extract is the most potent component of the plant with an IC_50_ value of 35.83 and 28.68 mg/L according to research on the DPPH antioxidant activity of plant components. According to the study the overall findings *R. officinalis* and *A. indica* could be a great source of antibacterial compounds. The potential application of these plant extracts as an antibacterial agent has broad ramifications and opens new research directions. Considering the information presented in this research, it can be clearly concluded that CYP450-mediated metabolic reactions are the basis of the relevant toxic effects reported. Further research on this plant to produce pharmaceuticals from natural sources was made possible by considerable biological discoveries and the proportion of active components discovered in *R. officinalis* and *A. indica* leaf extracts.

## Data availability statement

The original contributions presented in the study are included in the article/supplementary material. Further inquiries can be directed to the corresponding authors.

## Author contributions

Conceptualization, HA, MAl, MAb, MAh, AH, MAlj, SH, and YE-A. Validation, MAb, MAh, MAlj, SH, and YE-A. Formal analysis, SH and YE-A. Investigation, HA, MAl, MAb, MAh, AH, MAlj, RA, FG, SH, and YE-A. Data curation, MAl, SH, and YE-A. Writing-original draft preparation, MAl, SH, and YE-A. Writing-review and editing, HA, MAl, MAb, MAh, AH, MAlj, RA, FG, SH, and YE-A. All authors contributed to the article and approved the submitted version.
